# *Legionella pneumophila*-Induced NETs Do Not Bear LL-37 Peptides

**DOI:** 10.3390/microorganisms13102298

**Published:** 2025-10-03

**Authors:** Valeria Iliadi, Stefania Marti, Aikaterini Skeva, Konstantinos Marmanis, Theofani Tsavdaridou, Georgios Euthymiou, Eleni Tryfonopoulou, Dimitrios Themelidis, Athina Xanthopoulou, Katerina Chlichlia, Maria Koffa, Theocharis Konstantinidis, Maria Panopoulou

**Affiliations:** 1 Laboratory of Microbiology, Department of Medicine, Democritus University of Thrace, Dragana, 69100 Alexandroupolis, Greece; iliadi.valeria18@gmail.com (V.I.); stefaniamarti02@gmail.com (S.M.); skevakaterina@hotmail.com (A.S.); fanitsavdaridou@gmail.com (T.T.); euthymiougiorgos@gmail.com (G.E.); dthemelidis@gmail.com (D.T.); athina.xanthopulu@gmail.com (A.X.); mpanopou@med.duth.gr (M.P.); 2 Laboratory of Cellular and Molecular Biology, Department of Molecular Biology and Genetics, Democritus University of Thrace, Dragana, 69100 Alexandroupolis, Greece; kostasmarmanis@gmail.com (K.M.); mkoffa@mbg.duth.gr (M.K.); 3 Laboratory of Molecular Immunobiology, Department of Molecular Biology and Genetics, School of Health Sciences, Democritus University of Thrace, Dragana, 69100 Alexandroupolis, Greece; eltr.mailbox@gmail.com (E.T.); achlichl@mbg.duth.gr (K.C.)

**Keywords:** *Legionella pneumophila*, neutrophils, neutrophil extracellular traps NETs, interleukin (IL)-1β, LL-37

## Abstract

*Legionella pneumophila* (*L. pneumophila*) infection is characterized by a wide spectrum of manifestations, from influenza-like illness to life-threatening atypical pneumonia with multiorgan failure. The aim of our study was the assessment of in vitro and ex vivo neutrophil activation in *L. pneumophila* infections, as well as the role of neutrophils’ peptides such as LL-37 in infection. The ability of neutrophils to form ex vivo extracellular traps (NETs) in response to bacterial infection was examined by immunofluorescence. In parallel, patients’ sera, as well as opsonized standard *L. pneumophila* strains, were used for in vitro activation of neutrophils from healthy individuals. The serum levels of interleukins were assessed using the LEGENDplexTM Multi-Analyte Flow Assay Kit. Furthermore, citrullinated cf-DNA as a marker of neutrophil extracellular traps (NETs) was detected in the serum of patients with acute infection. It was demonstrated that neutrophils released NETs in vitro and ex vivo upon *L. pneumophila* (interaction in an autophagy-independent manner. Notably, IL-1b was detected on NETs, but an antimicrobial peptide LL-37 was absent. The lack of antimicrobial activity failed to inhibit bacterial proliferation. In addition, in vitro and ex vivo NETs formation was observed during the Clarithromycin treatment. Those NETs were decorated with bioactive antimicrobial peptide LL-37, which inhibits bacterial proliferation. The findings provide evidence that neutrophils release NETs in vitro and ex vivo by expressing the IL1β protein in them. The lack of expression of the antimicrobial peptide LL-37 on the NETs demonstrates the inability of the cells to inhibit proliferation, and consequently the elimination of *L. pneumophila*. Clarithromycin plays a dual role in the elimination.

## 1. Introduction

The *Legionella* spp. was first described in 1947 as a “rickettsia-like” microorganism [1], and the first outbreak was reported during the 58th annual convention of the American Legion in Philadelphia [2]. Years later, those strains were characterized as etiological agents of airborne infections, and the microorganism was named *Legionella pneumophila (L. pneumophila*) [3]. It is a small, Gram-Negative, aerobic, non-sporogenous, non-capsule-forming and motile bacillus that belongs to the family Legionellaceae [4,5]. More than 50 species of *Legionella* spp., and more than 70 distinct serogroups, have been identified [6]. *Legionella pneumophila* is the most common pathogenic species, with *L. pneumophila* serogroup 1 (Lp1) being one of the most commonly detected serotypes that causes outbreaks worldwide [7]. The bacterium is encountered in the environment, in nature as well as in man-made sources, such as air-conditioning systems and car cabin air filters [8,9]. Infection due to *L. pneumophila* is owed to inhalation of bacteria-contaminated aerosols [10]. The clinical manifestation of infection has two distinct forms: non-pneumonic, a milder febrile flu-like syndrome named Pontiac Fever, and a pneumonic form called Legionnaires’ disease (LD), a severe pneumonia with extrapulmonary manifestations and multiorgan failure, often leading to a life-threatening state and then death [11,12,13].

After inhalation, *L. pneumophila* arrives at alveolar space, where it enters into the alveolar macrophages. [14]. In addition, bacterial strains may penetrate alveolar epithelial cells by different Legionnaires’ virulence factors [15]. Both alveolar macrophages and epithelial cells, as well as the same microbes, initiate neutrophil chemotaxis to the alveolar space [16]. Neutrophils are the most abundant cell type of circulating leukocyte in human blood, and they migrate through chemotaxis to the site of infection, establishing the groundwork for an innate immune response [17,18]. At the site of infection, neutrophils carry out their antimicrobial activity through interrelated processes—phagocytosis, degranulation and the production of neutrophil extracellular traps (NETs) [19]. NETs are filamentous structures with scaffolds made from DNA, both nuclear and mitochondrial, and decorated by histones and cytoplasmatic proteins, such as neutrophil elastase, myeloperoxidase (MPO), cathepsin G, etc. [20]. NETs exhibit direct antimicrobial activity by bacterial trapping and immobilization, as well as due to antimicrobial peptides such as LL-37 [21,22].

The human cathelicidin, or LL-37, is an antimicrobial peptide. Strong activity against bacteria and viruses results from extracellular activation via serine protease 3 and kallikrein 5. By raising the permeability of bacterial cell walls, LL-37 shows direct bactericidal action; simultaneously, it shows indirect antibacterial effects by binding to and neutralizing bacterial endotoxins, including LPS [23]. Moreover, it can upset bacterial biofilms [22].

Considering all the above, the present study aimed to examine in vitro and ex vivo the role of neutrophils in *L. pneumophila* infection via NET release and further identification of proteins that may be implicated in the pathophysiology of LD infection.

## 2. Materials and Methods

### 2.1. Subjects

To carry out this study, 5 LD patients, as well as 5 (n = 5) healthy individuals (HI), or controls, were enrolled. Regarding the clinical manifestation, it is well recognized that the infection has two distinct forms: the non-pneumonic form, or Pontiac fever, and Legionnaires’ disease, a more severe form of infection which includes pneumonia. All our patients suffered from LD, and three of them had lobular pneumonia, without some extrapulmonary manifestations. Moreover, two patients’ clinical manifestations included extrapulmonary manifestations of LD, with diarrhea and abdominal pain, acute renal injury and elevation of cardiac enzymes (CPK). The study design complied with the Helsinki Declaration and had the approval of the Ethics Committee of the University General Hospital of Alexandroupolis, Greece (Ref. Number 32859/27 June 2025). Routine laboratory tests were performed on all subjects enrolled in this study (Table 1). For diagnosis of LD, a STANDARD F Legionella Ag FIA test system (Analyzer + Test device) for detecting *Legionella pneumophila* serogroups 1, 3, 5, 6 and 8 antigens via a urine sample was performed (SDBiosensor, Suwon-si, Republic οf Korea). The inclusion criteria for the LD group were a positive test for detecting *L. pneumophila* serogroups 1, 3, 5, 6 and 8 antigens via urine sample, and absence of other infectious etiology. The inclusion criterion for the HI group (controls) was absence of clinical manifestations of infection. Exclusion criteria included recent surgery, or hospital-acquired infection, except for LD.

### 2.2. Bacterial Strains

*Legionella pneumophila subsp. pneumophila* (ATCC 33152™) was used in this study. Initially, the microbial strain was cultivated onto buffered charcoal-yeast extract (BCYE) agar plates. Bacteria were collected and resuspended into sodium saline (0.9%NaCl) to adjust the turbidity of bacterial suspensions to 0.5 McFarland standards. Then, opsonization of bacteria was performed by incubating with serum from healthy donors for 30 min at 37 °C [24].

Bacterial strains, a clinical isolate of *P. aeruginosa*, were preserved in glycerol broth at −80 °C. Bacteria from an overnight culture on MacConkey agar were suspended in saline to an optical density of 0.5 McFarland, corresponding to a concentration of approximately 108 CFU/mL To specify the functionality (antimicrobial activity) of LL-37 peptides, bacterial strains of *P. aeruginosa* and *L. pneumophila* were cocultured with NETs structures, generated ex vivo from LD patients, or in vitro by clarithromycin [22,24].

### 2.3. Cell Types

#### 2.3.1. Neutrophil

Venous blood from healthy individuals (HI) and LD patients were obtained in heparinized blood collection tubes (BD Vacutainer^®^ Heparin Tubes, Becton, Dickinson and Company, Franklin Lakes, NJ, USA). Then, neutrophils were isolated by performing Histopaque double-gradient density centrifugation (11,191 and 10,771, Sigma-Aldrich, St. Louis, MO, USA), as previously reported [25]. The purity of the isolated cells’ type (neutrophils) exceeded 98%, as observed by May–Grünwald–Giemsa staining. Neutrophils were seeded and cultured in 24-well plates at a density of 1–2 × 10^5^ cells per well in RPMI.

#### 2.3.2. THP1 Cells

Cell culture

The human monocytic THP-1 cell line (ATCC TIB202; American Type Culture Collection, Rockville, MD, USA) was routinely cultured in a humidified CO_2_ incubator at 37 °C in RPMI 1640 medium, supplemented with 10% heat-inactivated fetal bovine serum, L-glutamine, 100 U/mL penicillin and 100 μg/mL streptomycin (Biosera, Boussens, France).

THP-1 differentiation

THP-1 cells were seeded at a density of 2.5 × 10^5^ cells/well in 24-well plates with sterile glass coverslips fully attached to the bottom of each well. Concomitantly, THP-1 monocytes were differentiated into macrophages with phorbol myristate acetate (PMA, Sigma-Aldrich, St. Louis, MO, USA) at a final concentration of 50 ng/mL at 37 °C with 5% CO_2_ for 48 h in a humidified atmosphere. After a 24 h resting period without PMA, THP-1 cells were treated with serum samples from HI and LD.

### 2.4. Stimulation and Inhibition Studies

#### 2.4.1. Neutrophils’ Stimulation and NET Generation

Isolated neutrophils from LD patients, HI neutrophils, were incubated at 37 °C with 5% CO_2_ at a density of 1–2 × 105 cells per well in Roswell Park Memorial Institute 1640 medium (RPMI-1640, Thermo Fisher Scientific, Waltham, MA, USA), supplemented with 2% v/v FBS. Subsequently, they were incubated for 3 h at 37 °C with 5% CO_2_.

In addition, to investigate the role of neutrophils in the LD, neutrophils from healthy individuals (HI) were stimulated with: (a) 5% serum obtained from LD patients, (b) control serum (untreated condition), (c) Clarithromycin (Anfarm Hellas, Kifissia, Greece) was dissolved at a concentration of 50 mg/mL per stock, (d) opsonized *L. pneumophila* at a concentration of ~10 bacteria as previously reported and (e) serum from Familial Mediterranean Fever (FMF) patient as positive control for IL-1β decorated NETs [22,24,25].

#### 2.4.2. Autophagy Study

For in vitro or ex vivo autophagy induction, samples were stimulated and incubated as described above and incubated for 90 min [22,25].

#### 2.4.3. NET Structures Generation and Isolation

For in vitro or ex vivo NET structures generation, 2 × 10^6^ HI neutrophils were cultured in a 6-well cell culture plate (SPL Life Sciences, Kyonggido, Republic of Korea) in RPMI, supplemented with 2% v/v heterologous healthy donor serum, and stimulated with serum from LD patients or clarithromycin. Following a 3 h incubation (37 °C, 5% CO_2_), the cell culture medium was removed, cells were washed once with pre-warmed RPMI and fresh prewarmed RPMI was added. The cell culture plate was vigorously agitated, and NET structures were isolated. HI neutrophils that were not stimulated with patient serum served as control (control NETs) [22,25].

### 2.5. Immunofluorescence

#### 2.5.1. Neutrophil Seeding

Peripheral blood neutrophils were seeded on poly-L-lysine-coated glass coverslips (Biocoat, New York, USA) in a 24-well cell culture plate (SPL Life Sciences, Kyonggi-do, Republic of Korea) and incubated for 3 h (37 °C, 5% CO_2_) to evaluate their NET release capacity and examine the NET protein profile. In addition to studying autophagy induction, samples were incubated for 90 min. Cells were fixed with 10% formaldehyde solution (Biognost, Zagreb, Croatia) for 15 min at room temperature. Nonspecific binding sites were blocked with 6% normal goat serum (Thermo Fisher Scientific, Waltham, MA, USA) in 1x PBS (blocking solution).

#### 2.5.2. Antibodies

Following this, samples were stained with a primary antibody solution, consisting of a rabbit polyclonal anti-human neutrophil elastase (NE) poyclonal antibody (1:300 dilution, OriGene Technologies GmbH, Herford, Germany), a mouse anti-human IL-1β monoclonal antibody (mAb) (1:300 dilution, R&D System, Minneapolis, MN, USA) and a mouse anti-human LL-37 mAb (1:300 dilution, Santa Cruz Biotechnology, Dallas, TX, USA) in blocking solution, for 1 h at room temperature (RT). A polyclonal gout anti-rabbit IgG AlexaFluor 647 antibody (Invitrogen, Waltham, MA, USA) or a polyclonal rabbit anti-mouse IgG AlexaFluor 488 antibody (Invitrogen, Waltham, MA, USA). Finally, DAPI solution (Sigma-Aldrich, St Louis, MO, USA) was used for DNA counterstaining [22,25].

For protein detection by immunofluorescence, the following antibodies were used: mouse monoclonal antibody anti-LL-37 protein (1/300 dilution; Santa Cruz), mouse anti-IL1β and rabbit polyclonal antibody antι-Neutrophil Elastase (NE) (1/300 dilution; OriGene Technologies GmbH, Herford, Germany).

#### 2.5.3. Autophagy

To study autophagy induction, samples were stained with anti-LC3b polyclonal antibody (1:100 dilution, OriGene Technologies GmbH, Herford, Germany), followed by a polyclonal anti-mouse IgG AlexaFluor488 antibody (Invitrogen, Waltham, MA, USA) as a secondary antibody. DNA was counterstained using DAPI [22]. In addition, to clarify, autophagy inhibition by Bafilomycin, and more specifically Bafilomycin A1 (BafA1), which inhibits the final stage of autophagy, was used.

#### 2.5.4. Visualization

Imaging was performed on a customized Andor Revolution Spinning Disk Confocal system (Yokogawa CSU-X1; Yokogawa, Tokyo, Japan) built around an Olympus IX81 (Olympus Shinjuku, Tokyo, Japan) with 20× 0.45NA air lens, 40× 0.95NA air lens or 100× 1.40NA oil lens (UPlanXApo; Olympus Shinjuku, Tokyo, Japan) and a digital camera (Andor Zyla 4.2 sCMOS; Andor Technology Ltd., Belfast, Northern Ireland) (CIBIT-Bioimaging Facility, MBG-DUTH). The system was controlled by Andor IQ3.6.5 software (Andor Technology). Images were acquired as z-stacks with selected optical sections every 1 μm, through the entire cell volume.

#### 2.5.5. Quantification

Quantification of NETs with PicoGreen dye was performed by fluorescence measurements on a multimode plate reader (EnSpire, PerkinElmer, Waltham, MA, USA) and presented as Relative fluorescence units (RFU).

### 2.6. RNA Extraction, cDNA Synthesis and qPCR

Neutrophils from LD patients, HI neutrophils stimulated with Lp serum, as well as with clarithromycin, were processed for RNA extraction, cDNA synthesis, and qPCR, as previously detailed [25,26]. To verify LL-37 expression in Lp-serum-treated neutrophils in vitro, expression of LL-37 (forward primer: 50TGGTGTCACTGCTACTG30, reverse primer: 50CATTGCGGTGGAGATTC30) was evaluated via qPCR. GAPDH (forward primer: 50GGGAAGCTTGTCATCAATGG30, reverse primer: 50CATCGCCCCACTTGATTTTG30) was utilized to normalize LL-37 expression, following the housekeeping gene method of normalization. qPCR was performed, and the 2-DDCt method was applied for data analysis [27].

### 2.7. Bead-Based Multiplex Immunoassay

The serum levels of interleukin (IL)-1β, IL-6, IL-10, IL-18, IL-23 and IL-33, were assessed using the LEGENDplexTM Multi-Analyte Flow Assay Kit (Biolegend, San Diego, CA, USA) [28,29]. The samples were prepared following the manufacturer’s instructions, and data acquisition was performed by the Attune NxT Flow Cytometer (Invitrogen by Thermo Fisher Scientific Inc.).

### 2.8. Statistical Analysis

For quantitative variables, age and interleukin levels, we calculated mean ± standard deviation (SD). Nonparametric tests (Mann–Whitney and Kruskal–Wallis) and the Chi-square (χ2) test were performed. The data for qualitative variables are presented as percentages. Statistical analysis was performed using IBM SPSS 26. Statistical significance was set to *p* < 0.05.

## 3. Results

### 3.1. Inflammatory Cytokines Are Detected in the Circulation of LD Patients

**Figure 1:**
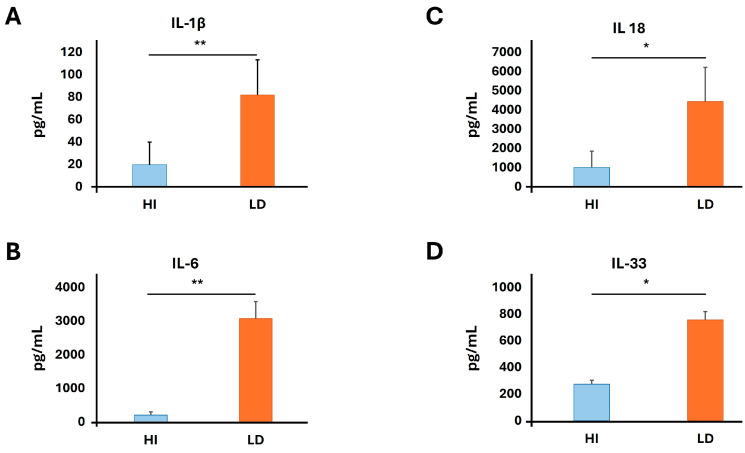
Proinflammatory cytokines in the circulation of LD patients. HI—healthy individuals, LD—Legionnaires’ disease, IL—interleukins. (**A**) IL-1β, (**B**) IL-6, (**C**) IL-18, (**D**) IL-33; * *p* < 0.001; ** *p* < 0.05.

Considering our recently published data, which demonstrate that *L. pneumophila*-induced NETs express a major proinflammatory cytokine, IL-1β [24], that plays a cornerstone role in multi-organ failure during the LD, we determined cytokines in the sera. To assess the inflammatory milieu of *L. pneumophila* infection, serum samples from patients were analyzed using a bead-based multiplex immunoassay. We observed that the levels of proinflammatory cytokines IL-1β, IL-6, IL-18 and IL-33 were elevated in LD patients compared to HI in a statistically significant manner (Figure 1). On the other hand, there was no difference between the patients and the control group for TNF-α, IL-8, IL-12p70, IL-17A, 40.05 ± 18.8 vs. 37.6 ± 19.4, *p* = 0.9, 77.8 ± 5.6 vs. 60.3 ± 7.6 *p* = 0.21, 27.8 ± 6.1 vs. 14.9 ± 7.8 *p* = 0.2 and 7.2 ± 1.9 vs. 4.4 ± 2.5, *p* = 0.12, respectively.

### 3.2. Neutrophils Release NETs as a Response to L. pneumophila Ex Vivo and In Vitro

**Figure 2:**
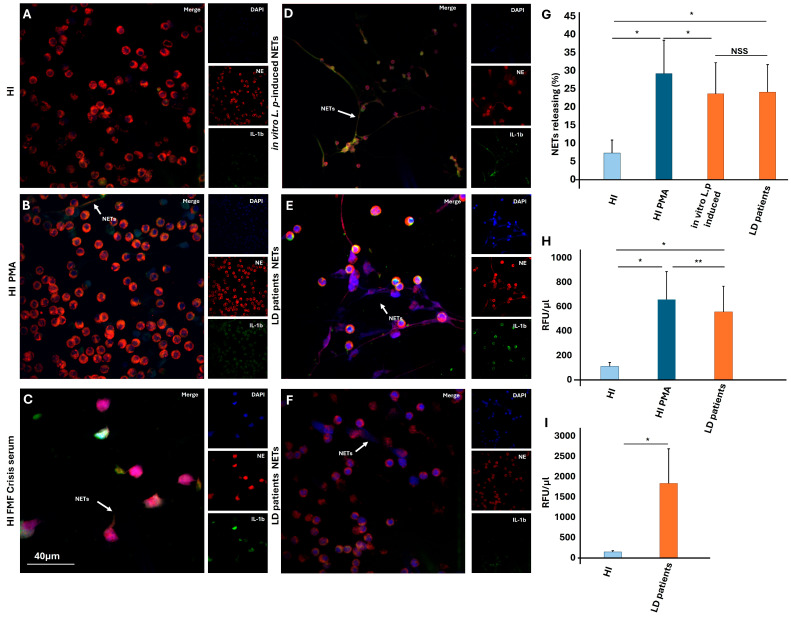
Fluorescence confocal microscopy images showing neutrophil elastase (NE) and IL-1β staining (blue: DAPI; red: NE; green: IL-1β). A representative example of 5 independent experiments is shown. (**A**) Untreated HI neutrophils; (**B**) In vitro isolated NETs, after stimulation of control neutrophils with PMA; (**C**) In vitro isolated NETs after stimulation of control neutrophils with FMF crisis; (**D**) In vitro isolated NETs, after stimulation of control neutrophils with serum from LD patients; (**E**) NETs from LD patients with severe pneumonia and extrapulmonary manifestations; (**F**) NETs from LD patients with pneumonia and without extrapulmonary manifestations. (**G**) percentage of NET formation; (**H**) NETs structures quantification by fluorescence-based method using the PicoGreen assay; and (**I**) Serum NETs structures quantification by fluorescence-based method using the PicoGreen assay in HI and LD serum samples. * *p* < 0.001; ** *p* < 0.05.

Considering the neutrophils’ ability to generate NETs as a mechanism to eliminate infection, we investigated whether activation of neutrophils during *L. pneumophila* infection releases NETs. We initially studied the ability of *L. pneumophila* to induce NETs formation ex vivo. NETs released by neutrophils from LD patients have been observed by immunofluorescence (Figure 2A–F), as well as the percentage of NETs formation (Figure 2G), and a fluorescence-based method using the PicoGreen assay (Figure 2H,I). Next, to support our ex vivo observations, neutrophils isolated from healthy individuals (HI) were stimulated with LD serum samples, representing the inflammatory disease microenvironment. This experiment highlights that the disease’s microenvironment can induce neutrophils to release NETs (Figure 2D). Furthermore, HI neutrophils were also pretreated with bacterial strains, as previously described [24]. We found that *L. pneumophila* pre-treated control neutrophils, as well as LD serum-treated cells, form NETs, as assessed via immunofluorescence microscopy. Several previous studies have demonstrated that potent inflammatory proteins expressed in NETs can determine the clinical manifestation of diseases [26,28,30,31]. In this context, to examine the proteinous components of NETs, and more specifically, the expression of IL-1β and correlation with disease severity, ex vivo and in vitro studies were performed (Figure 2D–F). In addition, HI neutrophils in vitro stimulated with FMF crisis serum were used as a positive control for IL-1β-bearing NETs (Figure 2C) [30]. We observe that the percentage of NETs released in LD patients with severe pneumonia and extrapulmonary manifestations was higher compared to patients with pneumonia and without extrapulmonary manifestations. Moreover, the IL-1β serum level was correlated with disease severity in a statistically significant manner 97.1 ± 24.8 vs. 32.1 ± 57.1, *p* = 0.003.

### 3.3. L. pneumophila-Induced NETs Are Unable to Express the Antimicrobial Peptide LL-37

**Figure 3:**
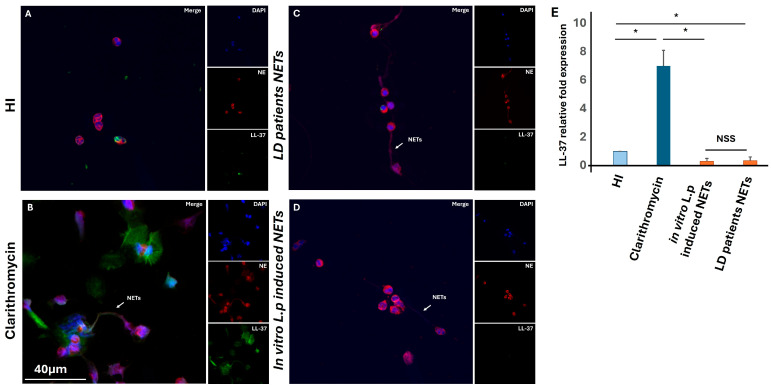
LL-37 expression on ex vivo and in vitro *L. pneumophila*-induced NETs. Fluorescence confocal microscopy images showing neutrophil elastase (NE) and LL-37 staining (blue: DAPI; red: NE; green: LL-37). A representative example of 5 independent experiments is shown. (**A**) Untreated HI neutrophils; (**B**) In vitro isolated NETs, after stimulation of control neutrophils with clarithromycin; (**C**) Isolated NETs, from LD patients; (**D**) In vitro isolated NETs, after stimulation of control neutrophils with serum from LD patients; (**E**) LL-37 expression in neutrophils, as assessed by RT-qPCR. * *p* < 0.001.

Considering that LL-37 is one of the potent neutrophils’ antimicrobial peptides with direct bactericidal properties that may prevent the spread of infection [23], we examined whether these NETs are decorated with the antimicrobial peptide LL-37 and whether these NET structures can inhibit bacterial growth in vitro. First, to detect the expression of LL-37 on the NETs, both ex vivo and in vitro studies were performed. Untreated neutrophils were exemplified as a negative control, while clarithromycin was used as a control for LL-37-positive NETs (Figure 3A,B). Surprisingly, we observed that ex vivo and in vitro *L*. *pneumophila*-induced NETs do not decorate with LL-37 (Figure 3C,D). Moreover, producing LL-37 poor NETs during the LD was also detected by a decrease in the mRNA LL-37 level in neutrophils, activated in vitro with both LD serum and ex vivo LD (Figure 3Ε).

**Figure 4:**
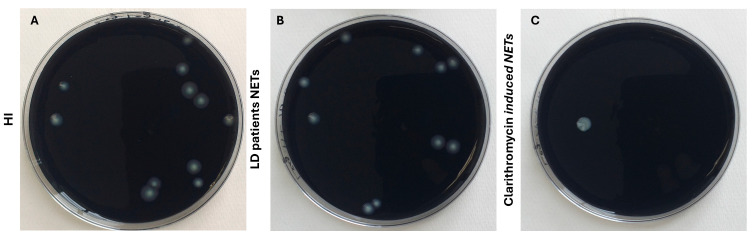
*L. pneumophila* proliferation is not inhibited in the presence of NET structures. (**A**) *L. pneumophila* cultures in BCYE plates with HI NETs structures without inhibition of proliferation; (**B**) *L. pneumophila* cultures in BCYE plates with LD patients NETs structures without inhibition of proliferation; (**C**) *L. pneumophila* cultures in BCYE plates with clarithromycin-induced NETs structures, with inhibition of proliferation.

Next, given the bactericidal properties of NETs, we examined whether in these LD NETs, poor antimicrobial peptide LL-37 can inhibit bacterial growth in vitro. As a positive control in this experiment, clarithromycin-induced NETs were used. We found that only clarithromycin-induced NETs and LL-37-decorated NETs were able to reduce bactericidal growth in vitro. On the contrary, LD NETs were unable to restrain *L. pneumophila* growth in vitro (Figure 4).

### 3.4. L. pneumophila-Induced NETs Are Unable to Activate Autophagy

**Figure 5:**
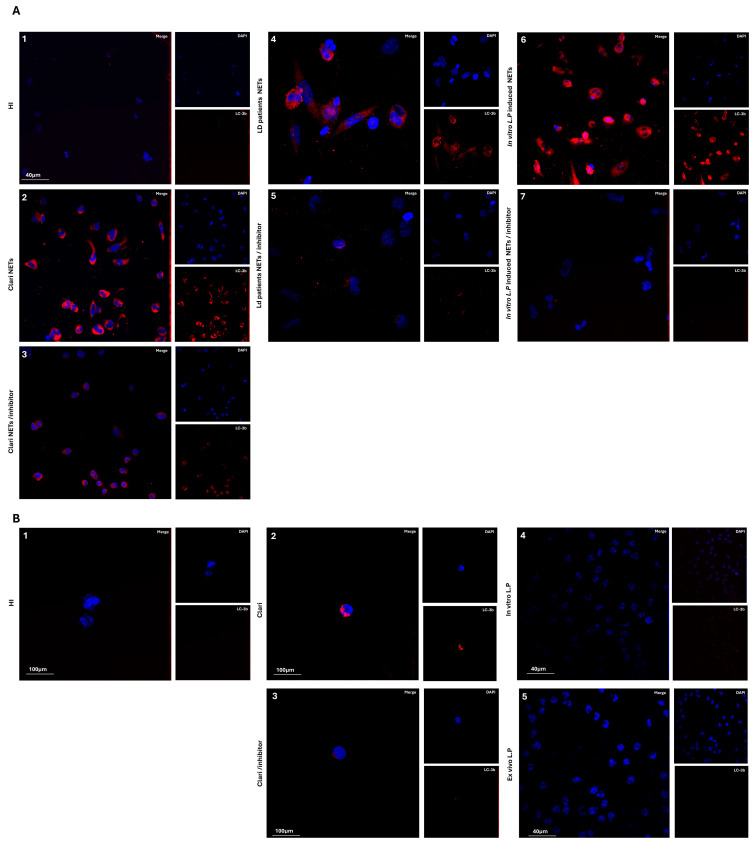
*L. pneumophila*-induced autophagy in different types of cells, macrophages and neutrophils. Fluorescence confocal microscopy images showing autophagy staining (blue: DAPI; red: LC3b). A representative example of 5 independent experiments is shown. (**A**) Study of autophagy on THP1 macrophages: (1) Untreated cells; (2) Cells stimulated with clarithromycin; (3) Cells stimulated with clarithromycin with inhibitors; (4) Cells stimulated with NETs from LD patients; (5) Cells stimulated with NETs from LD patients with inhibitors; (6) Cells stimulated with in vitro isolated NETs, after stimulation of control neutrophils with *L. pneumophila*; (7) Cells stimulated with in vitro isolated NETs, after stimulation of control neutrophils with *L. pneumophila* with inhibitors. (**B**) Study of autophagy on ex vivo or in vitro neutrophils: (1) Untreated HI neutrophils; (2) HI neutrophils with clarithromycin; (3) HI neutrophils with clarithromycin with inhibitors; (4) Neutrophils from LD patients; (5) In vitro isolated HI neutrophils stimulated with *L. pneumophila*.

Previous reports demonstrated that NET release triggered by *L. pneumophila* is ROS-dependent [24]. Next, given the role of autophagy in NETs release, we examined whether *L. pneumophila* utilizes autophagy to induce NET generation ex vivo or in vitro. Moreover, we examined whether autophagy utilization takes place in different types of cells, macrophages and neutrophils. To verify this, macrophages were stimulated with NETs structures, as assessed by LC3b staining. Surprisingly, we observed that *L. pneumophila* does not utilize autophagy to induce NET generation ex vivo and in vitro. In parallel, we found that *L. pneumophila*-generated NETs structures activate autophagy machinery in vitro in macrophages (Figure 5).

### 3.5. Dual Role of Macrolides in Legionella pneumophila Infection

It is well recognized that quinolones and macrolides have excellent activity against *Legionella* spp. strains in vitro and reach adequate inhibitory concentration within phagocytes (both macrophages and neutrophils) [32,33]. Previously, in our study, we demonstrated the in vitro and in vivo ability of macrolide antibiotics to induce NET formation. Moreover, we show that clarithromycin-induced NETs are decorated by the antimicrobial peptide LL-37, which is in its active form and can act against multidrug-resistant A. baumannii in vitro [22]. In this study, we observed that ex vivo and in vitro *L. pneumophila*-induced NETs do not decorate with LL-37 (Figure 3C,D). In addition, we found that LD NETs are unable to restrain *L. pneumophila* growth in vitro (Figure 4). Taking into account the antimicrobial effect of clarithromycin-induced NETs, both clarithromycin-sensitive strains of *L. pneumophila* and resistance strains of *P. aeruginosa* were analyzed. We confirm that LL-37-decorated NETs were able to reduce bactericidal growth in vitro, both in clarithromycin-sensitive strains of *L. pneumophila* and in resistance strains of *P. aeruginosa* (Figure S1).

## 4. Discussion

This article demonstrates that *L. pneumophila* induces NETs formation in vitro and ex vivo; however, the ability to be externalized on NETs antimicrobial peptides like LL-37 and their functionality is still unknown. In our study, LD-driven NETs were shown to be unadorned LL-37. The ability of microorganisms to induce neutrophil extracellular traps, which lack antimicrobial activity, was previously reported by Jhelum et al. Authors observe that the abundance of the antimicrobial peptide cathelicidin (CAMP) was lower on NETs induced by PVL-positive *S*. *aureus*, and as such, they were unable to kill *S. aureus* [34]. These data were in line with our results. In addition, the treatment of HI neutrophils with clarithromycin increases the levels of LL-37 on NETs, leading to significant in vitro induction of antibacterial activity of LL-37-bearing NETs on *L. pneumophila* strain, as much as *P. aeruginosa* strain. Previous studies have reported that LL-37 function is not limited to antimicrobial activity. When bound to DNA in the NETs, LL-37 protects DNA from degradation. Using immunofluorescence microscopy, Neumann et al., demonstrate that LL-37 decorated NETs are distinctly more resistant to *S. aureus* nuclease degradation [35]. Moreover, in our previous study, we demonstrated that upregulation of LL-37 on NETs improves the wound-healing capacity of fibroblasts [22,36]. Furthermore, its lipopolysaccharide (LPS)-neutralizing effect was reported [37].

Since neutrophils have been shown to express IL-1β under *L. pneumophila*. activation in vitro [24], we investigated its presence on *L. pneumophila* ex vivo-induced NETs. We demonstrate that during the LD, neutrophils produce NETs, which express a major proinflammatory cytokine IL-1β. It was shown that IL-1β expression depends on clinical manifestation. Moreover, it was shown that other cytokines of the IL-1 cytokine superfamily, IL-1β, IL-18 and IL-33, were also elevated. Similarly, a proinflammatory cytokine IL-6 was elevated in LD patients compared to HI, in a statistically significant manner. Our results are in line with previously reported data that show IL-18 or IL-33 elevation in *Legionella pneumophila* infection [38]. Rakebrandt et al. report a protective effect in subsequent heterologous challenges with *Legionella pneumophila* [38].

**Figure 6:**
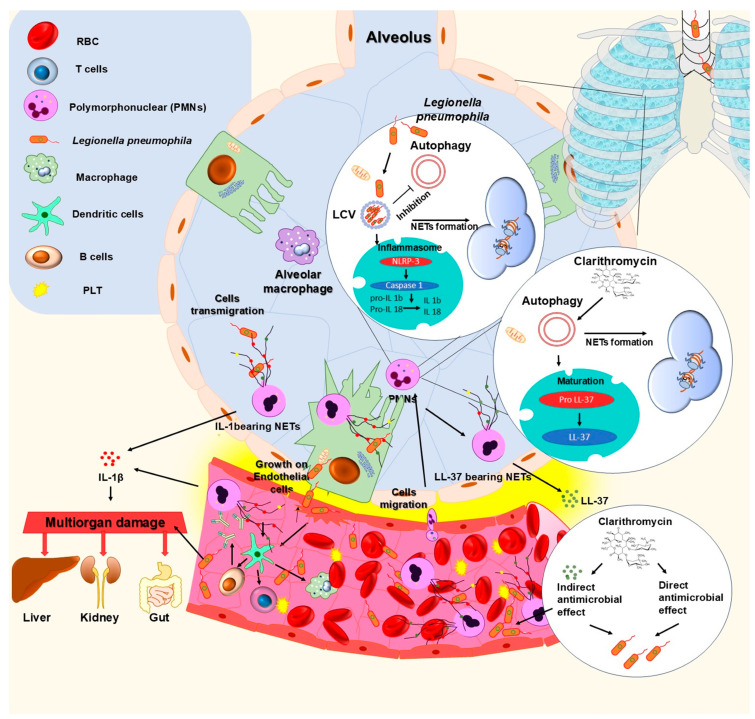
The pathophysiological mechanisms of LD.

Taking together the data presented in this article, we proposed the pathophysiological mechanisms of LD (Figure 6).

The bacteria, after the entry to the host cell, are within a vacuole (Legionella-containing vacuole—LCV). Afterwards, the intracellular replication of *Legionella* spp. is advanced. Furthermore, *L. pneumophila* binds via Mip protein to the collagen, which allows bacterial transmigration to the blood. In addition, *L. pneumophila* possesses autophagy inhibitory mechanisms in neutrophils. Further to this, *L. pneumophila* induces NETs formation, which are decorated with proinflammatory cytokines IL-1β that play a critical role in multiorgan damage in LD. On the other hand, the abundance of the antimicrobial peptide cathelicidin (CAMP), or LL-37, was lower on *L. pneumophila*-induced NETs. Finally, Clarithromycin-induced NETs bear LL-37. In this way, clarithromycin plays a dual role in Legionella infection.

Our study has limitations that we would like to mention. Initially, we had a small group of patients with LD. Consequently, we could not perform analysis with different disease phenotypes. Furthermore, we were unable to assess the quantitative determination of LL-37 in serum. Lastly, taking into account the role of IL-1β in inflammation and its elevated level in LD, we did not perform experiments with IL-1β inhibition. Hence, the next essential goal is to analyze our results in a large group of patients with LD, as well as the role of IL-1β inhibition by Kineret in the pathogenesis of multiorgan failure in LD.

## Figures and Tables

**Table 1:** Laboratory data on the studied population.

	LD n = 5	HI n = 5	*p*
Age	59.25 ± 16.6	50.4 ± 10.3	0.1
Male/Female	3/2	3/2	
Laboratory findings			
WBC (K/μL)	11.6 ± 8.9	6.4 ± 1.1	0.2
Neutrophil (%)	75.6 ± 14.2	69.7 ± 5.7	0.5
Lymphocytes (%)	17.8 ± 10	20.8 ± 3.9	0.6
Glu (mg/dL)	125.5 ± 21.9	108.25 ± 10.2	0.4
Urea (mg/dL)	66.5 ± 40.5	21.5 ± 3.7	<0.001
Creatinin (mg/dL)	2.3 ± 2.2	0.7 ± 0.1	0.2
eGFR (mL/min/1.7)	62.25 ± 43.2	103.25 ± 11.1	<0.001
SGOT (U/L)	83.5 ± 44.1	30.5 ± 17.8	0.02
SGPT (U/L)	141.7 ± 23.3	19.6 ± 15.7	0.01
LDH (U/L)	1398.6 ± 78.8	206.4 ± 25.5	0.27
CPK (U/L)	854.4 ± 890	82.5 ± 42.3	<0.001
TP (g/dL)	6.4 ± 0.3	6.7 ± 0.4	0.12
Albumin (g/dL)	3.5 ± 0.18	3.7 ± 0.27	0.03
γ-GT (U/L)	83.2 ± 29.6	24.4 ± 20.4	0.004
CRP (mg/dL)	24.03 ± 2.3	0.9 ± 1.1	<0.001
Procalcitonin (mg/dL)	113.6 ± 374.9	0.2 ± 0.8	<0.001

CPK, Creatine–phosphate–kinase; CRP, C-reactive protein; Glu fasting serum glucose levels; eGFR-estimated glomerular filtration rate; HI, healthy individuals or controls; LDH, lactate dehydrogenase; PCT, procalcitonin; SGPT, serum glutamate–pyruvate transaminase; SGPT, serum glutamic–pyruvic transaminase; TP, total protein; WBC, white blood cell. Calculation of eGFR was performed using the MDRD formula.

## Data Availability

The original contributions presented in this study are included in the article/Supplementary Material. Further inquiries can be directed to the corresponding author.

## Supplementary Materials

The following supporting information can be downloaded at: https://www.mdpi.com/article/10.3390/microorganisms13102298/s1, Figure S1: Pro-inflammatory cytokines in the circulation of LD patients Figure S2: *P. aeruginosa* proliferation in the presence of NET structures.

## Abbreviations

The following abbreviations are used in this manuscript:

HI	Healthy individuals
LD	Legionnaires’ disease
IL	Interleukins
NETs	Neutrophil Extracellular Traps
