# Supplementary material for: Legionella pneumophila-Induced NETs Do Not Bear LL-37 Peptides
Source: Microorganisms. 2025 Oct 3;13(10):2298. doi: 10.3390/microorganisms13102298 (PMC12566110; doi:10.3390/microorganisms13102298)
Supplement: Supplementary file 1 [file microorganisms-13-02298-s001.zip › microorganisms-3822341-supplementary.pdf]

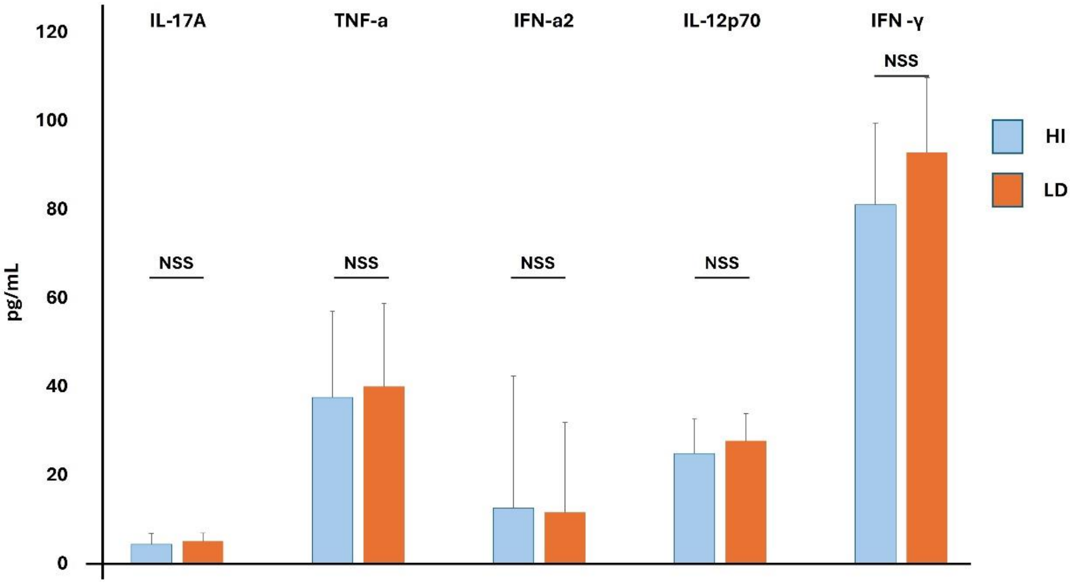

Figure S1. Pro-inflammatory cytokines in the circulation of LD patients.

HI - healthy individuals, LD Legionnaires' disease, IL interleukins

IL-17A: 4,4 ±2,5 vs 5,1±1.9, p=0.12; TNF-α: 37,6±19.4 vs 40.05±18.8, p= 0;19; IFN-α2: 12,52±14.8 vs 11,6±10.2, p=0.19; IL-12p70: 24,9±7.8 vs 27.8±6.1, p= 0.2; IFN-γ: 81.07±18.5 vs 92,88±16.9, p= 0.14.

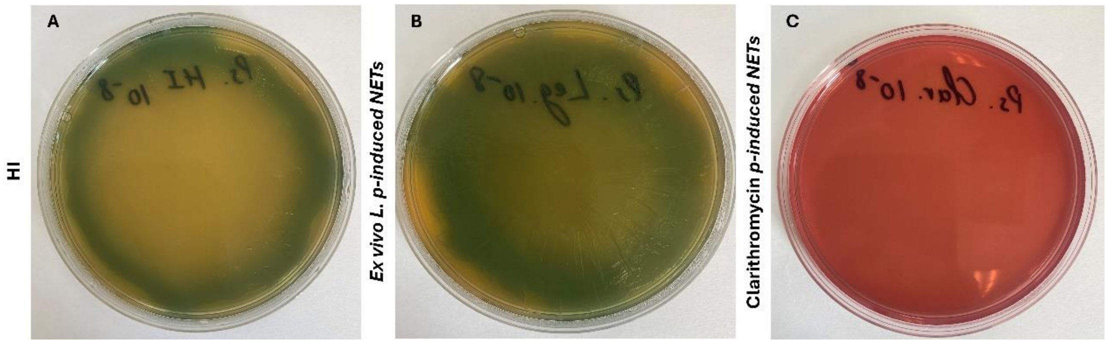

Figure S2. P. aeruginosa proliferation in the presence of NET structures. (A) *P. aeruginosa* cultures in MacConkey agar plates with HI NETs structures without inhibition of proliferation; (B) *P. aeruginosa* cultures in MacConkey agar plates with *ex vivo L. pneumophila* NETs structures without inhibition of proliferation; (C) *P. aeruginosa* cultures in MacConkey agar plates with clarithromycin-induced NETs structures, with inhibition of proliferation.
